# Counterion-insulated near-infrared dyes in biodegradable polymer nanoparticles for *in vivo* imaging†

**DOI:** 10.1039/d1na00649e

**Published:** 2021-11-18

**Authors:** Joanna Sobska, Bohdan Andreiuk, Ilya O. Aparin, Andreas Reisch, Wojciech Krezel, Andrey S. Klymchenko

**Affiliations:** Institute of Genetics and Molecular and Cellular Biology (IGBMC) – INSERM U1258, CNRS UMR-7104, University of Strasbourg 1, Rue Laurent Fries 67404 Illkirch France krezel@igbmc.fr; Laboratoire de Bioimagerie et Pathologies, UMR 7021 CNRS, Université de Strasbourg 74 Route du Rhin 67401 Illkirch France andrey.klymchenko@unistra.fr reisch@unistra.fr

## Abstract

Polymeric nanoparticles (NPs) are highly attractive for biomedical applications due to their potential biodegradability and capacity to encapsulate different loads, notably drugs and contrast agents. For *in vivo* optical bioimaging, NPs should operate in the near-infrared region (NIR) and exhibit stealth properties. In the present work, we applied the approach of ionic dye insulation with bulky hydrophobic counterions for encapsulation of near-infrared cyanine dyes (Cy5.5 and Cy7 bearing two octadecyl chains) into biodegradable polymer (PLGA) NPs. We found that at high dye loading (20–50 mM with respect to the polymer), the bulkiest fluorinated tetraphenylborate counterion minimized best the aggregation-caused quenching and improved fluorescence quantum yields of both NIR dyes, especially of Cy5.5. In addition, bulky counterions also enabled formation of small 40 nm polymeric NPs in contrast to smaller counterions. To provide them stealth properties, we prepared 40 nm dye-loaded PEGylated NPs through nanoprecipitation of synthetic PLGA–PEG block copolymer with the dye/counterion salt. The obtained NIR NPs loaded with Cy5.5 dye salt allowed *in vivo* imaging of wild-type mice with a good contrast after IV injection. Compared to the bare PLGA NPs, PLGA–PEG NPs exhibited significantly slower accumulation in the liver. Biodistribution studies confirmed the preferential accumulation in the liver, although PLGA and PLGA–PEG NPs could also be distributed in other organs, with the following tendency: liver > spleen > lungs > kidney > heart > testis > brain. Overall, the present work validated the counterion approach for encapsulation of NIR cyanine dyes into biodegradable polymer NPs bearing covalently attached PEG shell. Thus, we propose a simple and robust methodology for preparation of NIR fluorescent biodegradable polymer NPs, which could further improve the existing optical imaging for biomedical applications.

## Abstract 2

Bulky hydrophobic counterions were applied for encapsulation of near-infrared cyanine dyes into biodegradable polymer matrix with minimized self-quenching, yielding fluorescent nanoparticles with stealth PEG shell for *in vivo* imaging.

## Graphical abstract

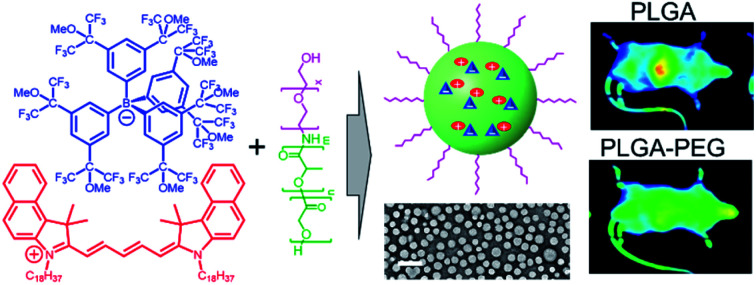

## Introduction

Polymeric nanoparticles (NPs) have attracted significant attention due to their capacity to encapsulate different loads, notably drugs and contrast agents.^1,2^ In particular, fluorescent polymeric nanoparticles are attractive as tools for biosensing and bioimaging.^3–5^ Among a large variety of *in vivo* optical imaging modalities,^6–9^*in vivo* fluorescence imaging is attractive because of simplicity of use, inexpensive detection setups and diversity of fluorescence imaging agents.^10,11^ However, *in vivo* fluorescence imaging requires imaging agents operating in the near-infrared region of the spectrum.^10–12^ There are two optical windows where the tissue presents better transparency and lower light scattering and light absorption: NIR-I (700–900 nm) and NIR-II (950–1500 nm). Although NIR-II is superior to NIR-I because of lower auto-fluorescence and light-scattering,^13,14^ NIR-I remains the dominant region for biomedical applications, because most imaging instruments operate in the NIR-I range using the FDA-approved indocyanine green as a NIR dye.^15–17^ Therefore, the development of polymeric NPs operating in the NIR-I remains an important task.

A number of approaches have been followed to prepare NIR-I polymeric nanoparticles. One is based on the use of conjugated polymers. Even though it is challenging to shift conjugated polymers to the NIR region, recent studies using conjugated donor–acceptor fluorophore systems showed remarkable examples exhibiting high brightness and good *in vivo* imaging contrast.^18,19^ However, these systems owing to their structures remain essentially non-biodegradable NPs. Another approach is to use biodegradable polymeric NPs^2,20^ as a nanocarrier of dyes. In this case, the biodegradable polymer PLGA,^20,21^ which is already approved in some medical applications, can be used as a particle matrix for loading a fluorescent dye. However, the problem is that at high dye loading conditions required to assure high brightness of NPs, the dyes tend to undergo aggregation-caused quenching (ACQ), due to formation of non-emissive H-aggregates.^22^ The problem of ACQ can be addressed by different approaches,^3^ including aggregation-induced emission (AIE),^23,24^ the use of bulky side groups,^25,26^ and bulky hydrophobic counterions.^27,28^ Previously, we have shown that bulky hydrophobic counterions are particularly effective to prevent ACQ in dye-loaded polymeric NPs.^27,29,30^ These counterions play a dual function: on one hand, they form hydrophobic ion pairs with cationic dyes to ensure their nearly quantitative encapsulation inside hydrophobic polymeric core of NPs prepared by nanoprecipitation.^29,31,32^ On the other hand, these bulky anions serve as spacers or insulators for encapsulated cationic dyes, thus preventing formation of non-emissive H-aggregates.^27,29,32^ Therefore, the NPs even at high loadings of cationic dyes paired with these counterions (up to 50 wt% with respect to polymer) displayed remarkably high fluorescence quantum yields (∼50%).^33^ These NPs can be up to 100-fold brighter than quantum dots of similar size and emission color.^27,34,35^ Moreover, the counterion also ensured short dye–dye distances in the polymer matrix that resulted in ultrafast excitation energy transfer,^27^ where thousands of loaded dye molecules could efficiently transfer energy to few energy acceptors generating a giant light-harvesting nanoantenna for single-molecule detection at ambient light.^36^ The universal concept of bulky counterions is applicable to both rhodamine and cyanine dyes, which enabled preparation of fluorescent NPs of any desired color in the visible range and notably developing a cell barcoding method for *in vitro* and *in vivo* applications.^30^ Despite high loading of dyes with their bulky counterions, PLGA NPs did not show significant cytotoxicity even at concentrations 10 times those used for bioimaging.^30^ Dye-loaded polymeric NPs have been successfully converted into biosensors for detection of nucleic acids with single-molecule sensitivity^33,35^ and also applied for single-particle tracking inside cells.^37,38^ However, *in vivo* application of this type of NPs have been limited so far to two-photon intravital microscopy using rhodamine-loaded NPs non-covalently coated with Pluronic surfactant.^39^ To adapt these NPs to the whole animal NIR imaging, the counterion approach should be applied to the encapsulation of near-infrared dyes into polymeric NPs. Earlier works showed that bulky anions could increase performance of NIR dyes of the cyanine family, but these works were done either on pure dye salts^40^ or on polymeric coatings at low dye loading.^41^ In addition, we intended to implement a covalent PEGylation strategy for these dye-loaded NPs, which has been proven to be fruitful to ensure stealth properties to polymeric NPs.^42–44^

In the present work, we applied the bulky counterion approach to the encapsulation of two near-infrared dyes based on Cy5.5 and Cy7 into NPs based on the biodegradable polymer PLGA. Evaluation of four counterions revealed that fluorinated tetraphenylborates can drastically increase fluorescence quantum yield of the encapsulated dyes by minimizing their ACQ. Moreover, they ensure formation of small NPs, in contrast to dye salts with the small inorganic anion chloride. Then, to provide PEGylation to these NPs, we synthesized PLGA–PEG block copolymer and applied it for the formulation of 40 nm NPs loaded with NIR dye Cy5.5. Optimized formulations based on PLGA and a PLGA–PEG were then tested for *in vivo* imaging of mice. Studying the biodistribution of these NIR particles showed that PEGylated PLGA NPs loaded with Cy5.5 with bulky counterions are promising systems for *in vivo* imaging.

## Materials and methods

Poly(d,l-lactide-*co*-glycolide) (PLGA, lactide 50 mol%, glycolide 50 mol%, acid terminated, *M*_n_ 25 300, PDI 1.3) was purchased from PolySciTech (Akina, Ref. AP082, Lot 70201AMS-A). The amino-terminated PEG NH_2_–PEG–OH (PEG3010) with molecular weight 3000 Da was purchased from Iris Biotech.

### Synthesis

Synthesis of dyes and their ion pairs with bulky counterions are described in ESI.†

### PLGA–PEG

300 mg of PLGA (0.012 mmol, 1 equiv. of COOH) were dissolved in anhydrous dichloromethane (2.5 mL). To this solution DIPEA (14 μL, 0.082 mmol, 7 equiv.) and HATU (25 mg, 0.066 mmol, 5.5 equiv.) were added as solutions in anhydrous dimethylformamide (total of 1.5 mL) under argon. 140 mg of NH_2_–PEG–OH (0.047 mmol, 4 equiv.) were dissolved in 1 mL of DMF and added to the reaction mixture. After stirring for 24 h at room temperature, part of the solvent was evaporated at 40 °C under reduced pressure. The obtained solution was precipitated in methanol. The precipitate was washed with methanol, redissolved in acetonitrile, and reprecipitated twice in methanol. After drying under a vacuum, 172 mg of a white solid were obtained (yield 52%). ^1^H NMR (400 MHz, CDCl_3_): *δ*/ppm 5.3–5.1 (m, 1H), 4.9–4.6 (m, 2H), 4.30 (m, 0.07H), 3.8–3.4 (m, 1.34H), 1.8–1.4 (m, 3H). Degree of modification 96%.

### Preparation of NPs

Stock solutions of polymers were prepared at a concentration of 10 g L^−1^ in acetonitrile. These were used to prepare acetonitrile solutions containing 2 to 4 g L^−1^ of polymer and the desired amount of dye salts. For particle preparation the solutions were quickly added to a 9-fold volume excess of phosphate buffer (20 mM, pH 7.4) and water, for PLGA and PLGA–PEG, respectively, under shaking (Thermomixer comfort, Eppendorf, 1000 rpm, at 21 °C). Directly afterwards, the solutions were either further diluted to the desired concentration in the aqueous phase, or the acetonitrile was removed through evaporation under reduced pressure (for *in vivo* applications).

### Characterization of NPs

#### Dynamic light scattering (DLS)

The sizes of the NPs were measured on a Zetasizer Nano series ZSP (Malvern Instruments S.A.). Each sample was measured 10 times with a run length of 10 s each. The volume average values were used, which are determined by the Zetasizer software (Malvern) based on Mie theory. Mean values give the average over at least three independent measurements, error bars correspond to standard error of the mean. Absorption and emission spectra were recorded on a Cary 4000 Scan ultraviolet-visible spectrophotometer (Varian) and on a Fluoromax 4 spectrofluorometer (Horiba Joban Yvon) equipped with a thermostated cell compartment, respectively. Quantum yields of Cy5.5-C18 and Cy7-C18 NPs were determined using methanolic solutions of DiD (QY = 0.33) and DiR (QY = 0.28) with optical density < 0.1 as references.

### Transmission electron microscopy

Solutions of NPs (5 μL) were deposited onto carbon-coated copper–rhodium electron microscopy grids following amylamine glow-discharge. They were then treated for 20 s with a 2% uranyl acetate solution for staining. The obtained grids were observed using a Tecnai F20 Twin transmission electron microscope (FEI Eindhoven Holland) operating at a voltage of 200 kV. Images (2048 pixels × 2048 pixels) were recorded using a US1000 camera (Gatan). After drying, the uranyl acetate remaining on the sample provides the contrast, often negative, but in some cases also in the form of a ring or shadow around the particles. Images were analyzed using the Fiji software. At least 200 particles per condition were analyzed.

### Animals

CD1 male mice were obtained from Charles River Laboratory and were imaged at 5–6 weeks of age. All animals were housed in the 12 : 12 light/dark cycle with food and water available *ad libitum*. The experiments were approved by local ethics committee (authorisation no. 2018030111543287) and accredited by the French Ministry for Superior Education and Research in accordance with the Directive of the European Union Council (2010/63/EU), and were carried in compliance with the guidelines of CNRS and the French Agricultural and Forestry Ministry (decree 87848).

### *In vivo* imaging

CD1 mice were anesthetized using inhalation anesthesia system with isoflurane and were intravenously (IV) injected with two types of nanoparticles: PLGA or PLGA–PEG loaded with 20 mM Cy5.5/F12-TPB (100 μL per mouse). Images were collected using Lumina XRMS imaging system (PerkinElmer) at different time points: 0 (prior to injection) and at 2, 10, 20, 30, 60, 90, 120, 180 minutes after injection. Mice were euthanized with CO_2_ directly after imaging and all organs (brain, lung, kidney, spleen, liver, testis, heart) were collected. Living mice and tissues were analyzed using Lumina XRMS imaging system (PerkinElmer) and the following filters were applied: excitation at 660 nm and emission at 710 nm. Additional non-treated mice were euthanized with CO_2_ and the same organs as above were collected and used as a negative control.

## Results and discussion

### Encapsulation of NIR dyes into PLGA NPs: role of counterion

**Fig. 1:**
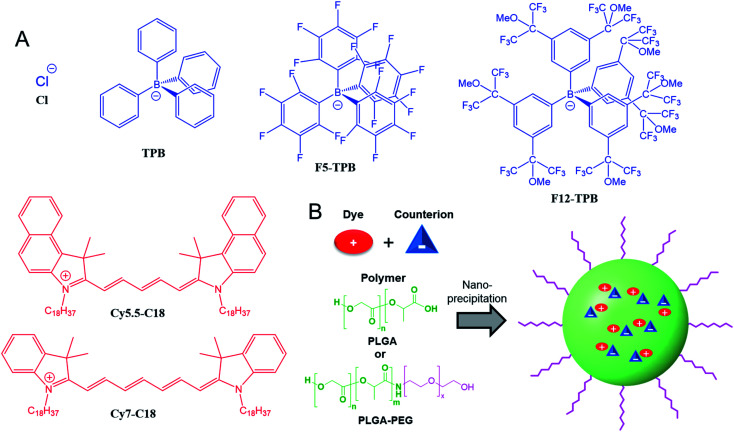
(A) NIR cyanine dyes (in red) and their counterions (blue) used in this study to prepare dye-loaded polymeric NPs. (B) Scheme of NPs preparation by nanoprecipitation of the polymer (PLGA or PLGA–PEG) with a cationic dye and its counterion.

**Fig. 2:**
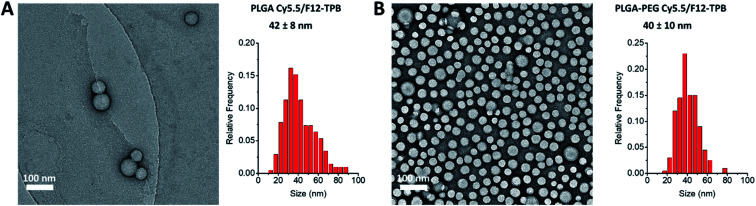
TEM images (left) and corresponding size distribution histograms (right) for PLGA (A) and PLGA–PEG (B) NPs loaded with 20 mM Cy5.5/F12-TPB dye. The values correspond to the average NP diameter, while errors correspond to the half-width at the half maximum. At least 200 NPs were analyzed per condition.

Cyanine NIR dyes Cy5.5-C18 and Cy7-C18 were selected for encapsulation into PLGA NPs (Fig. 1) because of their high hydrophobicity, cationic nature, and favorable optical properties. Four counterions were studied here: chloride (Cl), tetraphenylborate (TPB), tetrakis(pentafluorophenyl)borate (F5-TPB), and tetrakis[3,5-bis(1,1,1,3,3,3-hexafluoro-2-methoxy-2-propyl)phenyl]borate (F12-TPB). These salts were obtained in good yields (Table S1†) by a simple ion exchange followed by purification through column chromatography on silica gel. Thin layer chromatography (TLC) of the obtained salts revealed a strong effect of counterion on their retardation factor (*R*_f_). The chloride salts did not move at all on TLC in pure dichloromethane, whereas F5-TPB and F12-TPB salts showed the highest mobility (Fig. S1†). The *R*_f_ of TPB salts was intermediate, as their hydrophobicity is lower than those of the fluorinated TPBs. Three dye loadings of Cy5.5 and Cy7 dyes in the PLGA NPs were studied: 5, 20 and 50 mM with respect to the polymer. PLGA NPs were prepared by the nanoprecipitation method, previously described by us.^27,45^ An acetonitrile solution of the polymer and the dye at appropriate concentration was added rapidly to an aqueous phase under intense stirring, followed by a second dilution or evaporation of acetonitrile. At neutral pH, PLGA is negatively charged, which ensures formation of relatively small NPs of 40–50 nm.^27^ Indeed, according to dynamic light scattering (DLS) of PLGA NPs loaded with Cy7/TPB salts (Cy5.5 – NPs were not measured because of incompatibility with DLS), small sized NPs (46–60 nm) with low polydispersity (∼0.1, Table S2†) were observed. However, in the case of the chloride salt (Cy7/Cl), we observed a clear increase in the particle size with the dye loading, so that at 50 mM, very large aggregates of 780 nm size with relatively high polydispersity (0.26) were observed. According to our previous studies, this increase in size was systematically observed for different cationic dyes and it can be explained by the adsorption of non-encapsulated dye on the NPs surface leading to neutralization of its negative surface charge and further aggregation. By contrast, bulky tetraphenylborates ensure effective encapsulation of dyes preventing surface adsorption and thus favor formation of stable particles. Here, we show that this is valid also for a NIR cyanine dye. As we could not perform DLS of Cy5.5-loaded NPs, we studied by TEM a selected formulation of NPs loaded with the Cy5.5/F12-TPB salt at 20 mM. The observed NPs of round shape were of 42 nm diameter (Fig. 2), confirming that the counterion approach also allowed obtaining small Cy5.5-loaded NPs.

**Fig. 3:**
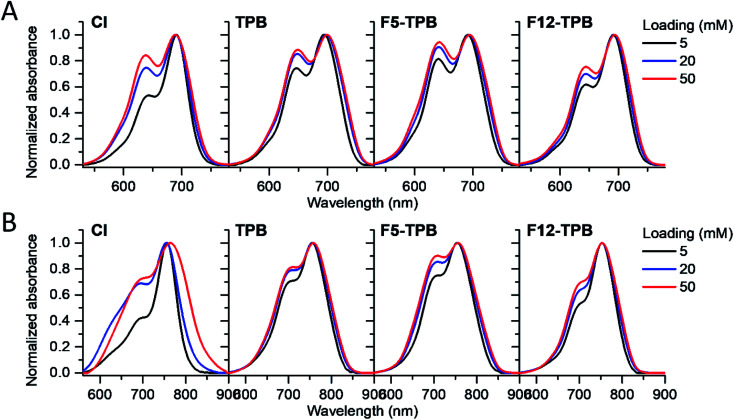
Normalized absorption spectra of PLGA NPs loaded at varied concentration (5, 20, or 50 mM with respect to polymer) of Cy5.5 (A) and Cy7 (B) dyes paired with different counterions.

Then, we characterized the optical properties of the obtained NPs using absorption and fluorescence spectroscopy. The absorption spectra of Cy5.5- and Cy7-loaded NPs showed an increase in the short-wavelength band intensity with loading (Fig. 3), indicating an increase in the dye aggregation at higher loadings. However, in case of TPB counterions, this change in the absorption spectrum was systematically much lower compared to those with chloride counterion and their bands were narrower, especially for Cy7 dye. Moreover, in case of F12-TPB, the short-wavelength band was systematically lower for both dyes at three studied loadings suggesting lower dye aggregation (Fig. 3). The effect of counterion was the most pronounced at the highest studied dye loading (50 mM, Fig. S2†), where the dyes were expected to have the strongest aggregation behavior. Thus, we can conclude that F12-TPB in particular decreases the aggregation of both Cy5.5 and Cy7 dyes in PLGA NPs.

**Fig. 4:**
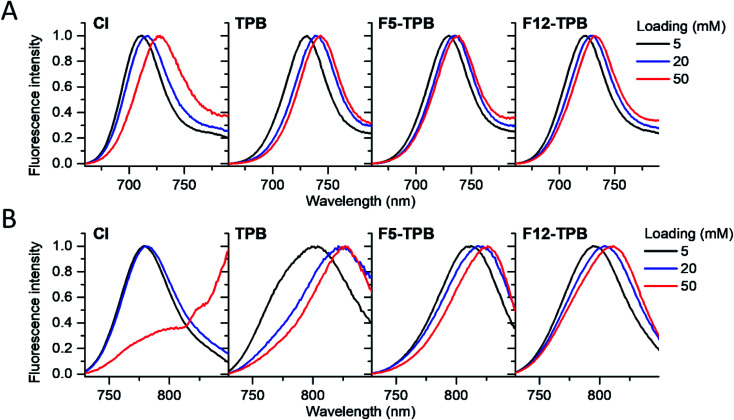
Normalized fluorescence spectra of PLGA NPs loaded at varied concentration (*vs.* polymer) with Cy5.5 (A) and Cy7 (B) dyes paired with different counterions.

In the fluorescence spectra, an increase in the dye loading in PLGA NPs systematically produced a red shift (Fig. 4 and Table 1). These shifts are in line with increased aggregation level of the dyes observed in the absorption spectra, as aggregation generally generates species of lower energy.^22,23^ Again, the strongest spectroscopic alternations due to the dye loading were observed for both dyes with chloride counterion, whereas the smallest effects were observed for F5-TPB and F12-TPB (Fig. 4). An increase in the dye loading also led to a rapid drop in the fluorescence quantum yield (QY, Table 1), suggesting the presence of aggregation-caused quenching. However, this drop was clearly less pronounced for the fluorinated TPBs F5-TPB and F12-TPB, so that at the highest dye loading, they showed the highest QY for both dyes. One should note that F12-TPB showed slightly higher QY values compared to F5-TPB, which is probably related to the larger size of the former anion. Indeed, the larger size could provide a better structural match with relatively large cyanine dyes, in line with our previous studies on cyanines operating in the visible spectral range.^30^ Moreover, Cy7 dyes showed much lower QY values compared to Cy5.5 dyes, especially at higher dye loadings. Thus, the counterion approach is less effective for the cyanine with the longest conjugation chain, which is probably related to its lower energy gap allowing more deactivation pathways in the aggregated state. Based on these formulations, we selected Cy5.5/F12-TPB at 20 mM, combining good QY with high loading, as the most suitable for *in vivo* studies in mice.

**Table 1:** Spectroscopic properties of PLGA NPs loaded with NIR dyes with different counterions at varied loading

Loading	Absorption maximum, nm			Fluorescence maximum, nm			Quantum yield, %		
	5 mM	20 mM	50 mM	5 mM	20 mM	50 mM	5 mM	20 mM	50 mM
Cy5.5/Cl	691	689	689	711	717	728	14.7 ± 0.1	1.72 ± 0.08	0.30 ± 0.01
Cy5.5/TPB	694	698	699	731	738	743	11.9 ± 0.1	1.81 ± 0.08	0.92 ± 0.01
Cy5.5/F5-TPB	691	693	694	730	736	739	12.5 ± 0.2	3.36 ± 0.01	1.75 ± 0.06
Cy5.5/F12-TPB	691	692	694	724	730	731	15.1 ± 0.6	4.94 ± 0.08	1.91 ± 0.14
Cy7/Cl	756	755	764	780	780	>850	8.3 ± 0.4	0.96 ± 0.10	0.10 ± 0.02
Cy7/TPB	756	757	758	804	821	827	1.1 ± 0.1	0.19 ± 0.01	0.09 ± 0.01
Cy7/F5-TPB	754	756	756	812	818	826	1.62 ± 0.03	0.30 ± 0.01	0.13 ± 0.01
Cy7/F12-TPB	752	753	753	795	804	811	4.55 ± 0.21	0.77 ± 0.01	0.33 ± 0.01

In order to apply these NPs *in vivo*, non-specific interactions at their surface should be minimized. To this end, we modified our NPs with polyethylene glycol (PEG), which is known to provide stealth behavior and prolong the circulation time in blood.^44,46^ This was achieved by using a PLGA–PEG block copolymer for the formulation of NPs.^47,48^ The block copolymer was obtained by coupling of PLGA to NH_2_–PEG–OH (3000 Da) using peptide chemistry. The obtained block copolymer was characterized by NMR, which suggested a modification degree of 96%. Using a similar nanoprecipitation protocol, we prepared PLGA–PEG NPs along with PLGA NPs loaded with Cy5.5/F12-TPB and Cy7/F12-TPB at 20 mM. TEM microscopy confirmed formation of NPs of *ca.* 40 nm size for both PLGA and PLGA–PEG (Fig. 2 and Table S3†). Small sized PLGA–PEG NPs could be also formulated with Cy7.5/F12-TPB (Table S3†). Nevertheless, only Cy5.5-loaded NPs were selected for *in vivo* imaging because of their significantly higher fluorescence quantum yields (Table 1). After 24 h of storage at room temperature in the buffer, only minor increase in the size and small decrease in the absorbance and fluorescence intensity were observed for both PLGA and PLGA–PEG NPs loaded with 20 mM Cy5.5/F12-TPB (Fig. S3†). Thus, within the timeframe of experiments, NPs preserve their basic characteristics.

**Fig. 5:**
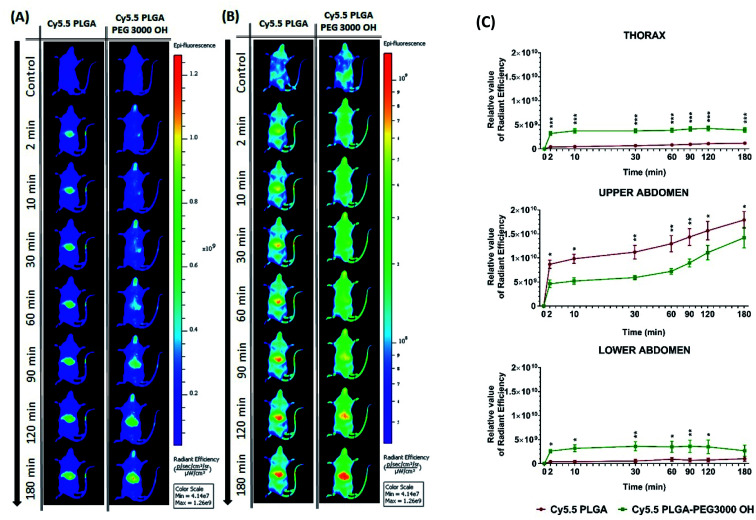
*In vivo* real-time imaging and biodistribution of nanoparticles. (A and B) CD1 mice were injected *via* tail vein with 100 μL of PLGA NPs or PLGA–PEG NPs loaded with 20 mM Cy5.5/F12-TPB and were analyzed with Lumina XRMS imaging system at different time points ((A) radiant efficiency scale, (B) logarithmic scale). (C) The relative value of the fluorescence signal in three different area of the animal's body: thorax (lungs), upper abdomen (liver), lower abdomen (intestine and bladder) at different time points (*n* = 4–5; *p < 0.05; **p < 0.01; ***p < 0.001). For area determination in the mice body see Fig. S4.†

Then, we characterized the behavior of our Cy5.5-loaded PLGA and PLGA–PEG NPs *in vivo* on wild-type mice. Mice were imaged at different time points with respect to IV injection of NPs using a Lumina XMRS NIR imaging setup (Fig. 5). According to the obtained images, already at the early post-injection times the signal of bare PLGA NPs appeared at the level of liver and further increased slowly over time (Fig. 5). By contrast, PLGA–PEG NPs showed much weaker fluorescence signal at the level of liver, but the signal grew steadily with time and then reached values close to those for PLGA NPs. Two-way ANOVA statistical analyses confirmed a significant effect of NPs PEGylation on the signal intensity in the thoracic region (*F*(1,6) = 55.59, *p* = 0.0003), upper abdomen corresponding to liver (*F*(1,6) = 9.46, *p* = 0.0218) and lower abdomen (*F*(1,6) = 6.403, *p* < 0.05) (Fig. 5 and S4†). Fisher's LSD *post hoc* analysis indicated that such differences reflect a significantly increased signal intensity in the thorax and lower abdomen area coming from PLGA–PEG nanoparticles. This localization difference between two types of NPs was recorded over the entire 3 hour imaging period in all designed body regions with the largest reordered change in the thoracic region (*p* < 0.001 for all recorded time points). The thorax contains the lungs (Fig. S4†), which is highly vascularized organ containing considerable amount of the overall blood volume, and this may be the reason for a significant increase in signal in this area. However, the highest quantities of both types of NPs were detected was the liver corresponding to the upper abdomen (Fig. 5 and S4†). There we have detected a higher presence of bare nanoparticles at time points between 30 and 90 minutes after injection of Cy5.5 PLGA NPs (*p* < 0.005). These results suggest that PEGylated NPs accumulate in the liver much slower than bare PLGA NPs, which is probably related to significantly longer circulation time of PLGA–PEG NPs. They confirm the crucial role of the PEG shell to ensure stealth properties of the polymeric NPs. Moreover, these data show that our encapsulation approach using Cy5.5 with bulky counterion F12-TPB ensures sufficient particle brightness to provide good contrast in NIR imaging. Indeed, the signal from the liver region of the injected mice after 180 min was 4 times higher than that of the control (non-injected) mice.

**Fig. 6:**
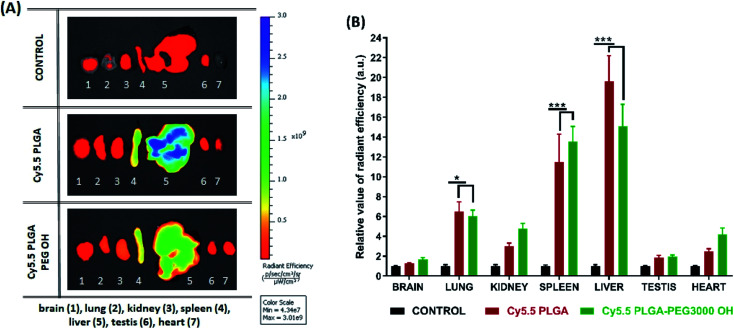
Biodistribution and accumulation of nanoparticles in organs. (A) *Ex vivo* fluorescence imaging of organs (brain, lung, kidney, spleen, liver, testis, heart). CD1 mice were sacrificed at 3 hours after intravenous injection with nanoparticles: Cy5.5 PLGA and Cy5.5 PLGA–PEG OH. Imaging was performed using Lumina XRMS imaging system. (B) Relative value of radiant efficiency has been normalized to an autofluorescence signal from organs of uninjected animals (*n* = 4, *p < 0.05; **p < 0.01; ***p < 0.001).

Further analysis of organs from mice dissected after 3 h of imaging confirmed accumulation of NPs in certain organs. Indeed, when compared to the control group, PLGA NPs with and without PEG displayed significant fluorescence reflecting their accumulation in the liver (*p* < 0.001), spleen (*p* < 0.001) and lung (*p* < 0.01), but not in the kidney, heart, testes and brain. Comparison of the intensity of such fluorescent signal between organs pointed to accumulation with following preference: liver > spleen > lung and other organs as indicated by respective statistical differences (*p* < 0.01 for liver *vs.* spleen, *p* < 0.001 for spleen *vs.* lung and absence of statistical differences between lung and any of the other organs). Slightly lower signal from the liver was observed for PEGylated NPs (Fig. 6), in line with *in vivo* imaging data analysis for the upper abdomen (Fig. 5C), although such difference was not significant most probably due to the late time-point of organ analysis. Overall, this analysis shows that PLGA–PEG NPs can distribute in different organs of healthy mice after IV injection, and suggest that PEGylation increases the time spent in the blood circulation.

## Conclusions

With the aim to prepare fluorescent polymeric NPs operating in the NIR spectral region, we studied encapsulation of ion pairs of NIR cyanine dyes with bulky hydrophobic counterions in PLGA NPs. So far, the counterion approach has been shown to effectively prevent ACQ effects for dyes operating in the visible region, but it has not yet been applied for near-infrared dyes in polymeric NPs. Here, we tested two cyanines Cy5.5 and Cy7 with four counterions: small inorganic chloride and bulky hydrophobic tetraphenylborates which differ in size and level of fluorination. We found that at high loading (20–50 mM *vs.* polymer) of the dyes, the two fluorinated counterions decreased the dye aggregation and improved fluorescence quantum yields of both NIR dyes. Among the studied tetraphenylborates, the bulkiest fluorinated counterion was the most efficient against ACQ. However, it should be noted that the dye with the longest absorption/emission wavelengths (Cy7) showed the lowest quantum yields and the effect of the counterion was less pronounced. In addition, bulky counterions also ensured formation of small polymeric NPs, in contrast to those loaded with dyes and small inorganic chloride. In order to test these NPs *in vivo*, we prepared PEGylated NPs based on synthetic PLGA–PEG and Cy5.5 dye with fluorinated TPB counterion (F12-TPB). Thus, the bulky counterion approach of dye loading is also compatible with NPs prepared by nanoprecipitation from amphiphilic block co-polymers. The prepared NIR NPs allowed *in vivo* imaging of wild-type mice with a good contrast after IV injection. Remarkably, PLGA–PEG NPs exhibited significantly slower accumulation in the liver compared to the bare NPs, probably because the stealth PEG shell ensures longer circulation time. Biodistribution studies confirmed the preferential accumulation in the liver, although PLGA and PLGA–PEG NPs could also distribute in other organs, with the following tendency: liver > spleen > lungs > kidney > heart > testis > brain. Overall, the present work validated the counterion approach for encapsulation of NIR cyanine dyes into polymeric NPs, allowing preparation of NIR NPs with relatively good quantum yields and high dye loading. This study also shows suitability of our approach for preparation of PEGylated PLGA NPs for *in vivo* NIR imaging applications. The PEGylated NPs with extended circulation time will be of particular interest for specific targeting and imaging of tumors using specific ligands, including antibodies, which will be a subject of a dedicated study. We expect that the bulky counterion approach, validated here for NIR dyes, will help in preparation of new NIR fluorescent nanomaterials and their further applications *in vivo*.

## Conflicts of interest

There are no conflicts to declare.

## Supplementary Material

NA-004-D1NA00649E-s001
