# Supplementary material for: Counterion-insulated near-infrared dyes in biodegradable polymer nanoparticles for in vivo imaging
Source: Nanoscale Adv. 2021 Nov 18;4(1):39–48. doi: 10.1039/d1na00649e (PMC8691417; doi:10.1039/d1na00649e)
Supplement: NA-004-D1NA00649E-s001 [file NA-004-D1NA00649E-s001.pdf]

Electronic Supplementary Material (ESI) for Nanoscale Advances.
This journal is © The Royal Society of Chemistry 2021

Electronic supplementary information

Counterion-insulated near-infrared dyes in biodegradable polymer nanoparticles for in vivo imaging

Joanna Sobska,[1,§] Bohdan Andreiuk,[2,§] Ilya O. Aparin,[2] Andreas Reisch,[2,*] Wojciech Krezel,[1,*] Andrey S. Klymchenko[2,*]

[1] *Institute of Genetics and Molecular and Cellular Biology (IGBMC) - INSERM U1258; CNRS UMR-7104, University of Strasbourg, 1, rue Laurent Fries, 67404 Illkirch, France*

[2]*Laboratoire de Bioimagerie et Pathologies, UMR 7021 CNRS, Université de Strasbourg, 74 route du Rhin, 67401, Illkirch, France*

[§] These authors contributed equally to this work.

*Corresponding authors: andrey.klymchenko@unistra.fr; krezel@igbmc.fr; reisch@unistra.fr

Synthesis of dyes

Synthesis of Cy5.5 chloride

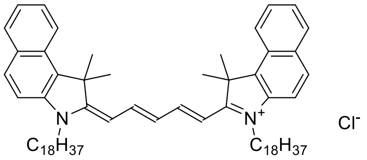

1,1,2-trimethyl-3-octadecyl-1H-benzo[e]indol-3-ium iodide (1 eq., 2 g, 3.39 mmol) was placed into a 50 mL round-bottom flask, after what 10 mL of dry pyridine were added and solution was heated to boiling. Then, 1,1,3,3-tetramethoxypropane (1.5 eq., 0.835 g, 0.838 mL, 5.09 mmol) was added to the reaction mixture and left stirring under reflux for 3 h. After cooling down to room temperature solvent was evaporated under vacuum. 50 mL of dichloromethane were added to the obtained residue. The solution was washed with 1 N HCl (x3), then with brine and water. The crude product was purified by flash column chromatography on silica gel using ethyl acetate/dichloromethane (9:1) mixture as eluent. Obtained yield was 76% (2.8 g). ^{1}H NMR (400.13 MHz, $CDCl_3$) δ 8.20 (t, *J* = 13.0 Hz, 2H), 8.16 (d, *J* = 8.4 Hz, 2H), 7.93 (d, *J* = 9.0 Hz, 4H), 7.64 (m, 2H), 7.48 (m, 2H), 7.34 (d, *J* = 8.9 Hz, 2H), 6.60 (t, *J* = 12.4 Hz, 1H), 6.18 (d, *J* = 13.8 Hz, 2H), 4.11 (t, *J* = 7.4 Hz, 4H), 2.02 (s, 12H), 1.86 (m, 4H), 1.42-1.50 (m, 4H), 1.35-1.42 (m, 4H), 1.19-1.34 (m, 52H), 0.88 (m, 6H); ^{13}C NMR (100.61 MHz, $CDCl_3$) δ 174.33, 152.74, 139.31, 134.11, 131.80, 130.49, 129.91, 128.21, 127.87, 125.46, 125.12, 122.32, 110.30, 102.80, 51.22, 44.38, 31.89, 29.68, 29.66, 29.63, 29.58, 29.53, 29.42, 29.33, 29.31, 27.62, 27.47, 26.91, 22.66, 14.08; HRMS (ESI) m/z $[M-Cl]^+$ calcd for $C_{69}H_{103}N_2^+$, 959.8121; found 959.8164.

Synthesis of Cy7 chloride

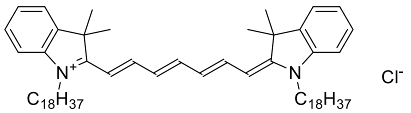

2,3,3-trimethyl-1-octadecyl-3H-indol-1-ium iodide (2.5 eq., 1000 mg, 1.85 mmol) and glutaconaldehydedianil hydrochloride (1 eq., 211 mg, 0.741 mmol) were added to 10 ml of dry pyridine, afterwards 1 ml of acetic anhydride was added and the mixture was heated at 50 °C under vigorous stirring. After the reaction completion pyridine was removed by rotary evaporation. The residue was dissolved in DCM and washed with 10% HCl 2 times and water once. DCM layer was collected, dried over sodium sulphate overnight, filtered from sodium sulfate and evaporated. Crude product was purified using gradient column chromatography with 99/1 to 9/1 DCM/MeOH mixture as eluent and obtained the product (1183 mg, 1.17 mmol, 63 %). ^{1}H NMR (400.13 MHz, DMSO-d6) δ 7.90 (m, 3H), 7.35 (m, 4H), 7.20 (m, 2H), 7.04 (m, 2H), 6.65 (t, *J* = 12.1 Hz, 2H), 6.22 (d, *J* = 12.9 Hz, 2H), 4.01 (m, 4H), 1.79 (m, 4H), 1.71 (s, 12H), 1.41-1.48 (m, 4H), 1.33-1.41 (m, 4H), 1.21-1.31 (m, 52H), 0.87 (m, 6H); ^{13}C NMR (100.61 MHz, $CDCl_3$) δ 171.44, 151.71, 142.23, 141.15, 128.45, 126.17, 124.73, 110.18, 103.67, 49.13, 44.45, 31.86, 29.64, 29.60, 29.56, 29.51, 29.39, 29.29, 28.06, 27.37, 26.95, 22.62, 14.06; HRMS (ESI) m/z $[M-Cl^-]^+$ calcd for $C_{63}H_{101}N_2^+$, 885.7965; found 885.8002.

General procedure of synthesis of ionic pairs

10 mg of Cy5.5 or Cy7 chloride were dissolved in 1 ml of dry DCM and 3-10 equivalents of the respective counterion were added to the mixture. The mixture was sonicated for 10s and the conversion was verified by TLC using DCM/MeOH 95/5 as eluent. Afterwards the mixture as purified by column chromatography using DCM/MeOH 95/5 as eluent to afford the product in 74 to 96% yield.

See the experimental details in the table below.

Table S1. Experimental conditions for synthesis of dye-counterion ion pairs.

Ion pair	Counterion added, eq	Product yield, %
Cy5.5/TPB	3	78
Cy5.5/F5-TPB	5	84
Cy5.5/F12	10	96
Cy7/TPB	3	74
Cy7/F5-TPB	5	88
Cy7/F12-TPB	10	93

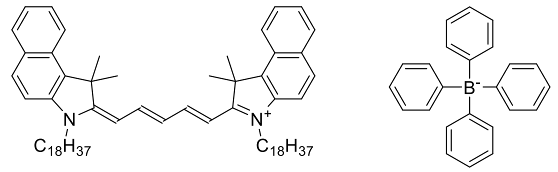

Cy5.5/TPB. ^{1}H NMR (400.13 MHz, $CDCl_3$) δ 8.08 (d, *J* = 8.5 Hz, 2H), 7.96 (d, *J* = 8.5 Hz, 2H), 7.93 (d, *J* = 8.9 Hz, 2H), 7.77 (t, *J* = 13.1 Hz, 2H), 7.63 (t, *J* = 7.2 Hz, 2H), 7.45-7.52 (m, 10H), 7.24 (d, *J* = 8.9 Hz, 2H), 7.04 (t, *J* = 7.3 Hz, 8H), 6.88 (m, 4H), 6.02 (t, *J* = 12.5 Hz, 1H), 5.91 (d, *J* = 13.6 Hz, 2H), 3.75 (t, *J* = 7.3 Hz, 4H), 1.95 (s, 12H), 1.73 (m, 4H), 1.28-1.38 (m, 8H), 1.23-1.28 (m, 52H), 0.88 (m, 6H); HRMS (ESI) m/z $[M-C_{24}H_{20}B^-]^+$ calcd for $C_{69}H_{103}N_2^+$, 959.8121; found 959.8168; HRMS (ESI) m/z $[M-C_{69}H_{103}N_2]^-$ calcd for $C_{24}H_{20}B^-$, 319.1658; found 319.1657.

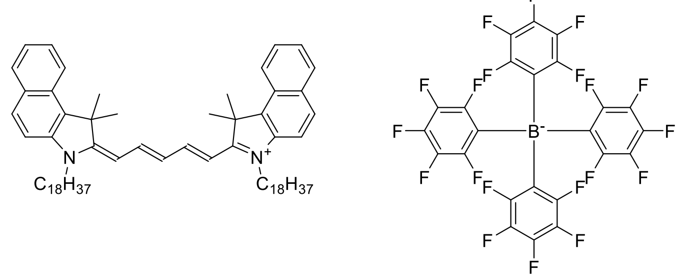

Cy5.5/F5-TPB. ^{1}H NMR (400.13 MHz, $CDCl_3$) δ 8.11 (d, *J* = 8.4 Hz, 2H), 7.94-7.97 (m, 4H), 7.88 (t, *J* = 13.1 Hz, 2H), 7.65 (m, 2H), 7.52 (m, 2H), 7.36 (br. s, 4H), 7.33 (d, *J* = 8.9 Hz, 2H), 6.20 (t, *J* = 12.4 Hz, 1H), 6.00 (d, *J* = 13.7 Hz, 2H), 4.04 (t, *J* = 7.5 Hz, 4H), 1.97 (s, 12H), 1.84 (m, 4H), 1.34-1.46 (m, 8H), 1.22-1.34 (m, 52H), 0.88 (m, 6H); ^{19}F NMR (376.50 MHz, $CDCl_3$) δ -132.26 (d, *J* = 10.2 Hz, 8F), -162.98 (t, *J* = 20.6 Hz, 4F), - 166.58 (t, *J* = 18.1 Hz, 8F). HRMS (ESI) m/z $[M-C_{24}F_{20}B^-]^+$ calcd for $C_{69}H_{103}N_2^+$, 959.8121; found 959.8164; HRMS (ESI) m/z $[M-C_{69}H_{103}N_2]^-$ calcd for $C_{24}F_{20}B^-$, 678.9774; found 678.9791.

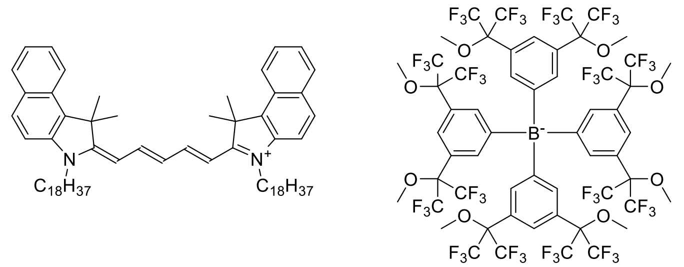

Cy5.5/F12-TPB. ^{1}H NMR (400.13 MHz, $CDCl_3$) δ 8.11 (d, *J* = 8.4 Hz, 2H), 7.99 (d, *J* = 8.7 Hz, 2H), 7.92 (t, *J* = 13.1 Hz, 2H), 7.67 (m, 2H), 7.54 (m, 2H), 7.51 (br. s, 8H), 7.36 (br. s, 4H), 7.34 (m, 2H), 6.52 (t, *J* = 12.4 Hz, 1H), 6.08 (d, *J* = 13.7 Hz, 2H), 4.08 (t, *J* = 7.6 Hz, 4H), 3.25 (s, 24H), 2.02 (s, 12H), 1.88 (m, 4H), 1.37-1.51 (m, 8H), 1.24-1.34 (m, 52H), 0.88 (m, 6H); ^{19}F NMR (376.50 MHz, $CDCl_3$) δ -71.33 (s, 48F); HRMS (ESI) m/z $[M-C_{56}H_{36}BF_{48}O_8^-]^+$ calcd for $C_{69}H_{103}N_2^+$, 959.8121; found 959.8165; HRMS (ESI) m/z $[M-C_{69}H_{103}N_2]^-$ calcd for $C_{56}H_{36}BF_{48}O_8^-$, 1759.1737; found 1759.1782.

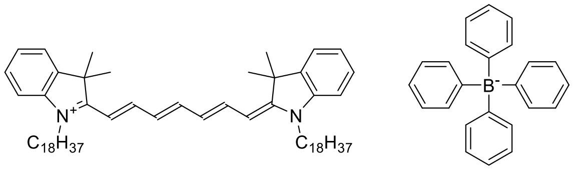

Cy7/TPB. ^{1}H NMR (400.13 MHz, DMSO-d6) δ 7.58 (t, *J* = 13.1 Hz, 2H), 7.46 (m, 8H), 7.31-7.38 (m, 4H), 7.22 (m, 2H), 7.14 (t, *J* = 12.6 Hz, 1H), 7.05 (t, *J* = 7.4 Hz, 8H), 6.96 (d, *J* = 7.9 Hz, 2H), 6.90 (m, 4H), 6.28 (t, *J* = 12.7 Hz, 2H), 5.88 (d, *J* = 13.7 Hz, 2H), 3.71 (t, *J* = 7.5 Hz, 4H), 1.70 (m, 4H), 1.60 (s, 12H), 1.31-1.38 (m, 8H), 1.23-1.30 (m, 52H), 0.89 (m, 6H); HRMS (ESI) m/z $[M-C_{24}H_{20}B^-]^+$ calcd for $C_{69}H_{103}N_2^+$, 885.7965; found 885.8005; HRMS (ESI) m/z $[M-C_{63}H_{101}N_2]^-$ calcd for $C_{24}H_{20}B^-$, 319.1658; found 319.1659.

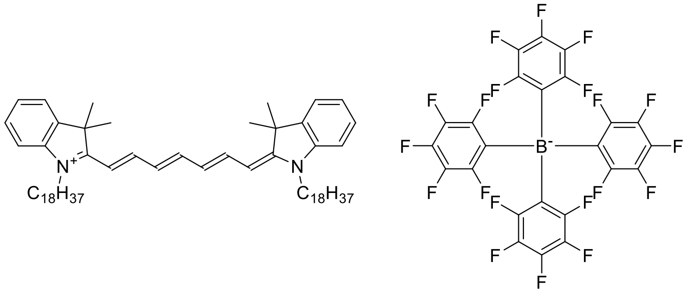

Cy7/F5-TPB. ^{1}H NMR (400.13 MHz, DMSO-d6) δ 7.66 (t, *J* = 13.1 Hz, 2H), 7.35-7.40 (m, 4H), 7.24 (t, *J* = 7.4 Hz, 2H), 7.04 (d, *J* = 7.9 Hz, 2H), 6.32 (t, *J* = 12.7 Hz, 2H), 5.99 (d, *J* = 13.7 Hz, 2H), 3.90 (t, *J* = 7.6 Hz, 4H), 1.79 (m, 4H), 1.63 (s, 12H), 1.30-1.46 (m, 8H), 1.21-1.30 (m, 52H), 0.88 (m, 6H); ^{19}F NMR (376.50 MHz, $CDCl_3$) δ -132.30 (d, *J* = 10.6 Hz, 8F), -163.11 (t, *J* = 20.6 Hz, 4F), - 166.70 (t, *J* = 18.2 Hz, 8F); HRMS (ESI) m/z $[M-C_{24}F_{20}B^-]^+$ calcd for $C_{63}H_{101}N_2^+$, 885.7965; found 885.8004; HRMS (ESI) m/z $[M-C_{63}H_{101}N_2]^-$ calcd for $C_{24}F_{20}B^-$, 678.9774; found 678.9771.

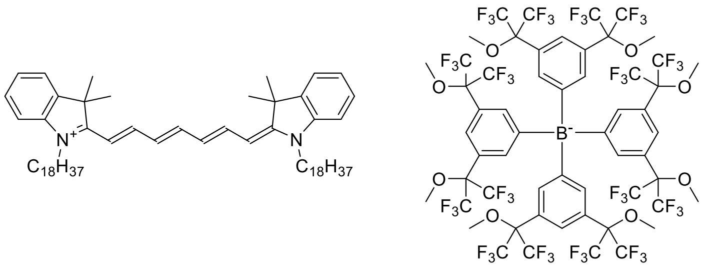

Cy7/F12-TPB. ^{1}H NMR (400.13 MHz, $CDCl_3$) δ 7.72 (t, *J* = 13.1 Hz, 2H), 7.51 (m, 8H), 7.32-7.42 (m, 9H), 7.27 (m, 2H), 7.07 (d, *J* = 7.9 Hz, 2H), 6.44 (t, *J* = 12.7 Hz, 2H), 6.03 (d, *J* = 13.6 Hz, 2H), 3.92 (t, *J* = 7.6 Hz, 4H), 3.25 (s, 24H), 1.80 (m, 4H), 1.66 (s, 12H), 1.35-1.45 (m, 8H), 1.22-1.34 (m, 52H), 0.88 (m, 6H); ^{19}F NMR (376.50 MHz, $CDCl_3$) δ -71.32 (s, 48F); HRMS (ESI) m/z $[M-C_{56}H_{36}BF_{48}O_8^-]^+$ calcd for $C_{63}H_{101}N_2^+$, 885.7965; found 885.8008; HRMS (ESI) m/z $[M-C_{63}H_{101}N_2]^-$ calcd for $C_{56}H_{36}BF_{48}O_8^-$, 1759.1737; found 1759.1757.

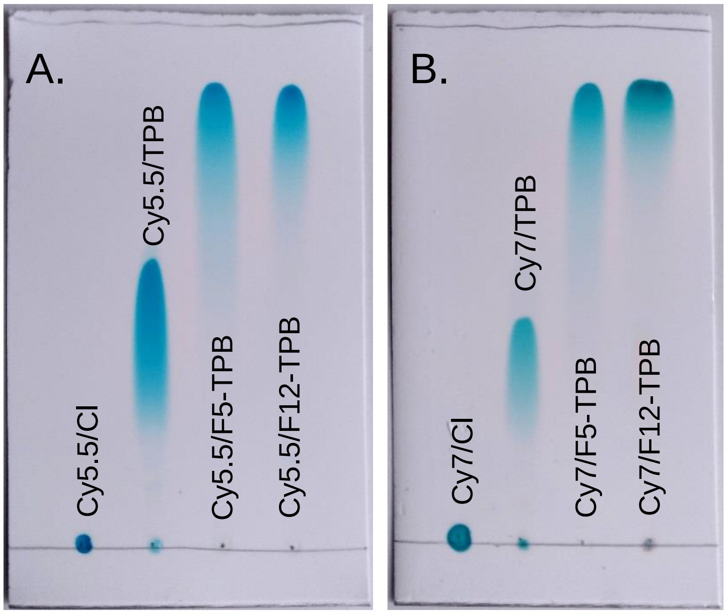

Figure S1. Representation of difference in mobility for four various ion forms of Cy5.5 (A.) and Cy7 (B.) dyes. Rf: (Cy5.5/Cl) - 0, (Cy5.5/TPB) – 0.49, (Cy5.5/F5-TPB) – 0.82, (Cy5.5/F12-TPB) – 0.82, (Cy7/Cl) - 0, (Cy7/TPB) – 0.39, (Cy7/F5-TPB) – 0.82, (Cy7/F12-TPB) – 0.88. Aluminum TLC plates pre-coated with SiO_2 (Merck) eluted with pure DCM.

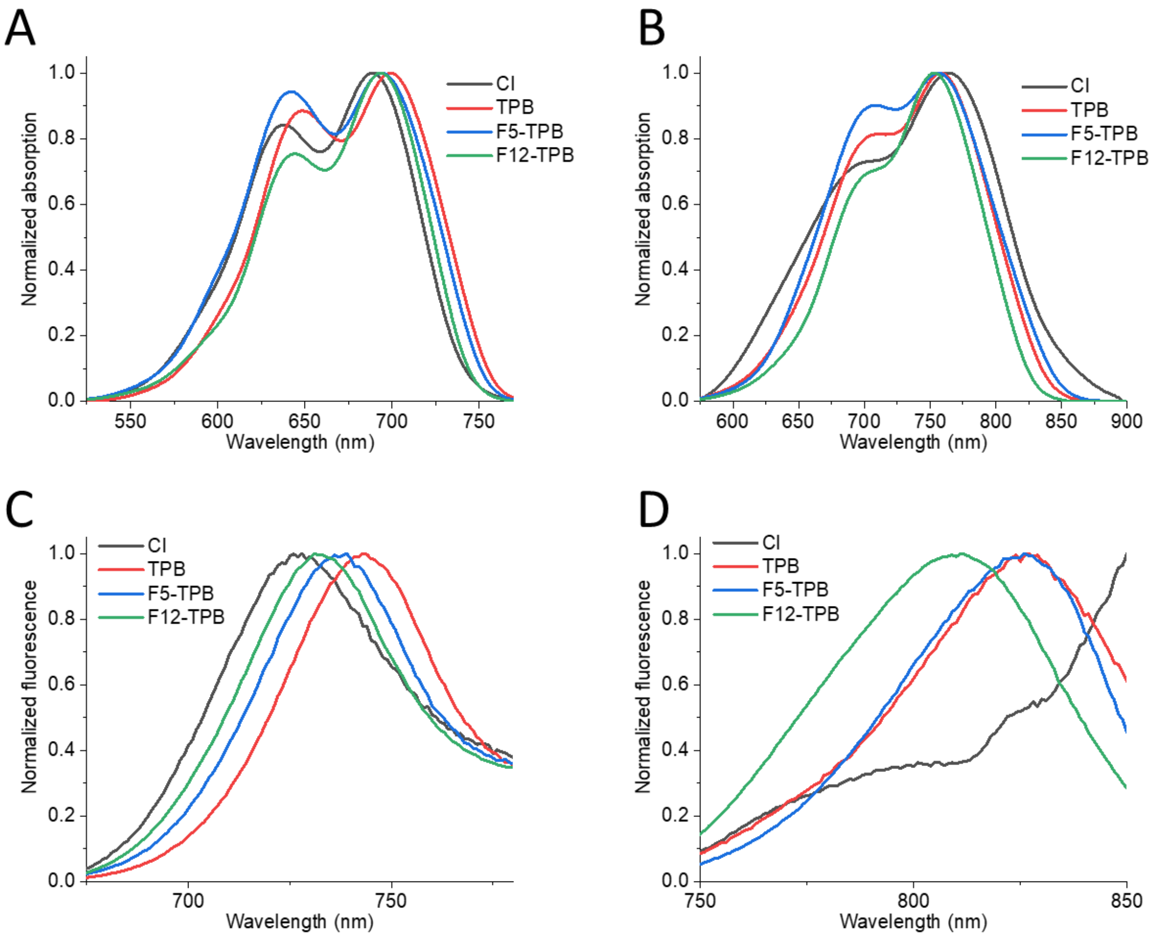

Figure S2. Absorption (A,B) and fluorescence (C,D) spectra PLGA NPs loaded at 50 mM concentration (vs polymer) with Cy5.5 and Cy7 dyes paired with different counterions.

Table S2. DLS data on PLGA NPs loaded with Cy7 and different counterions.[a]

Loading, mM	Counterion	Size (nm)	SD (nm)	PDI
**5**	Cl	54	2	0.13
	TPB	49	3	0.1
	F5-TPB	46	3	0.1
	F12-TPB	48	1	0.12
**20**	Cl	89	3	0.09
	TPB	51	1	0.07
	F5-TPB	51	2	0.12
	F12-TPB	53	2	0.14
**50**	Cl	780	70	0.26
	TPB	60	1	0.08
	F5-TPB	54	1	0.14
	F12-TPB	54	2	0.04

[a] Statistics by volume was used in the data analysis. SD is standard deviation of the mean (n = 3) and PDI is the polydispersity index.

Table S3. Sizes of PLGA and PLGA-PEG NPs loaded with Cy5.5 and Cy7.[a]

Polymer	Dye	Size (nm)	PDI
PLGA	Cy5.5	42 ± 8[b]	-
PLGA-PEG	Cy5.5	40 ± 10[b]	-
PLGA	Cy7.5	41 ± 2[c]	0.17
PLGA-PEG	Cy7.5	46 ± 2[c]	0.15

[a] Dye loading 20 mM vs polymer. [b] TEM data, average over > 200 particles, error gives width at half maximum. [c] DLS data, number average, error gives standard deviation over 3 independent formulations.

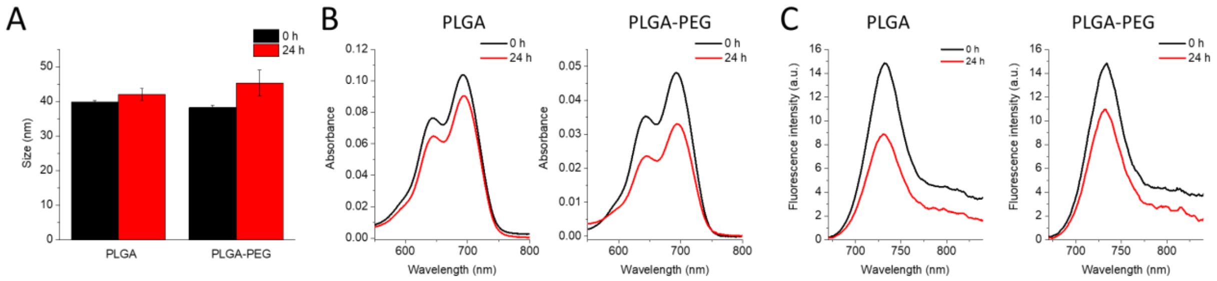

Figure S3. Evaluation of NPs stability in terms of size (A), absorption (B) and fluorescence (C) properties. PLGA and PLGA-PEG NPs loaded with 20 mM Cy5.5/F12-TPB with respect to the polymer. The samples were prepared and stored in 20 mM phosphate buffer (pH 7.4). Size (A) was obtained by DLS. Excitation wavelength (C) was 660 nm. Samples were either measured immediately after preparation (0 h) or after 24 h of storage in darkness at room temperature.

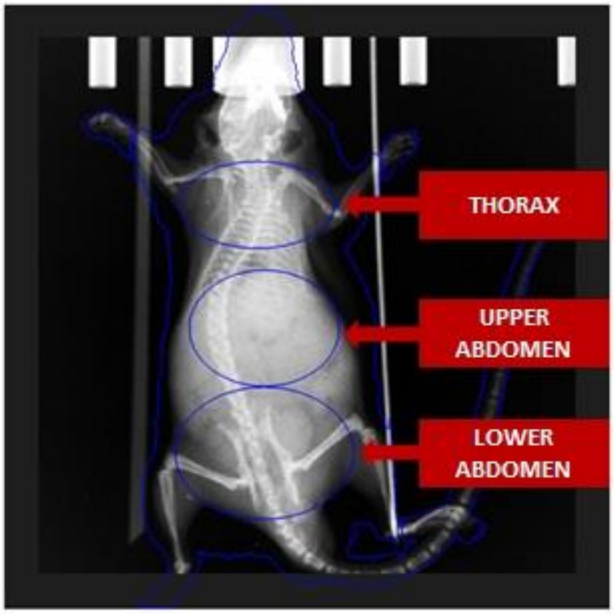

Figure S4. Selected areas, which were analyzed after the nanoparticles injection: thorax containing lung and heart, upper abdomen containing mainly liver and spleen and lower abdomen containing bladder. The areas are selected based on the position of the bones to minimize false positive measurements.

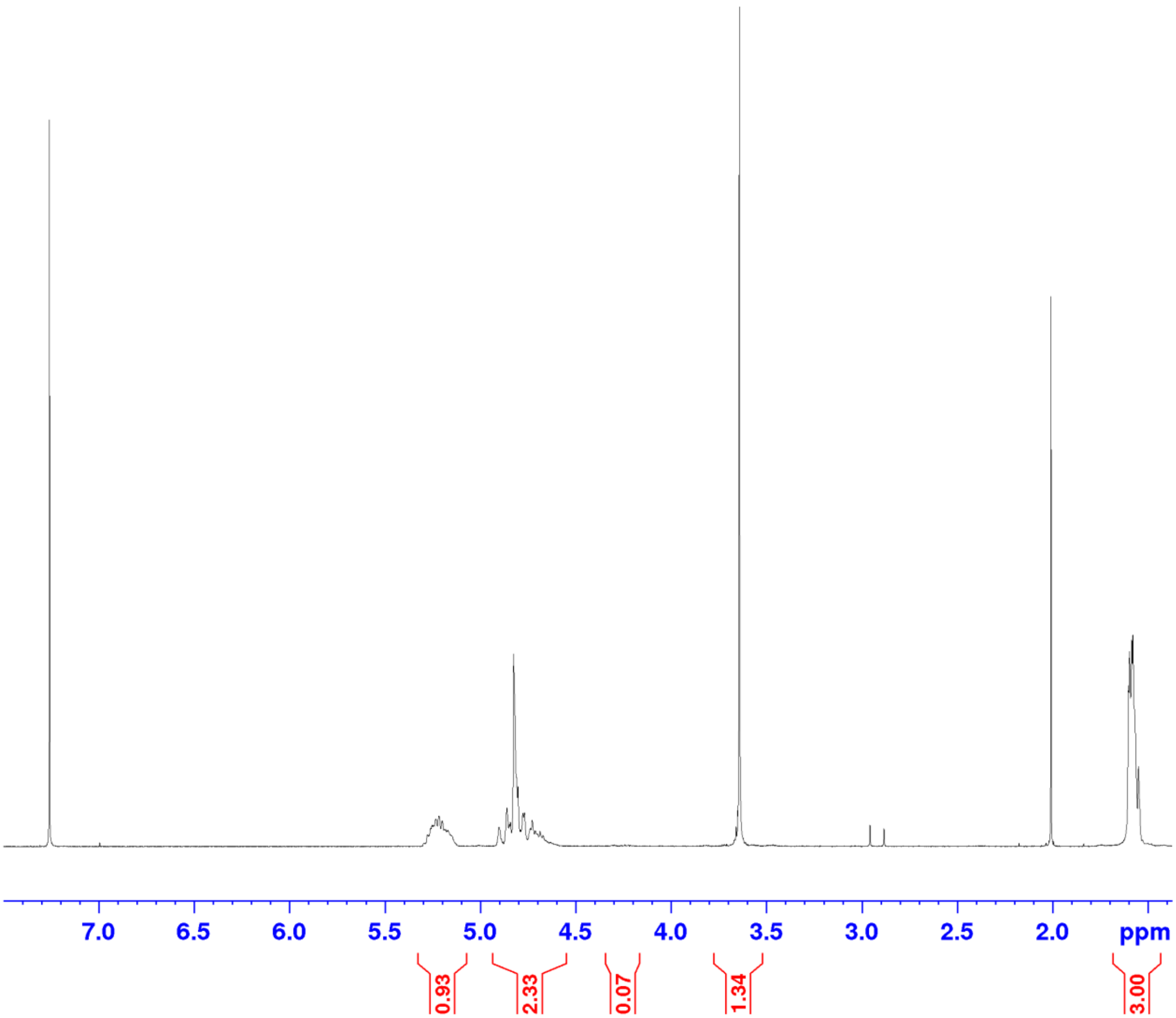

^{1}H-NMR spectrum of PLGA-PEG in $CDCl_3$.

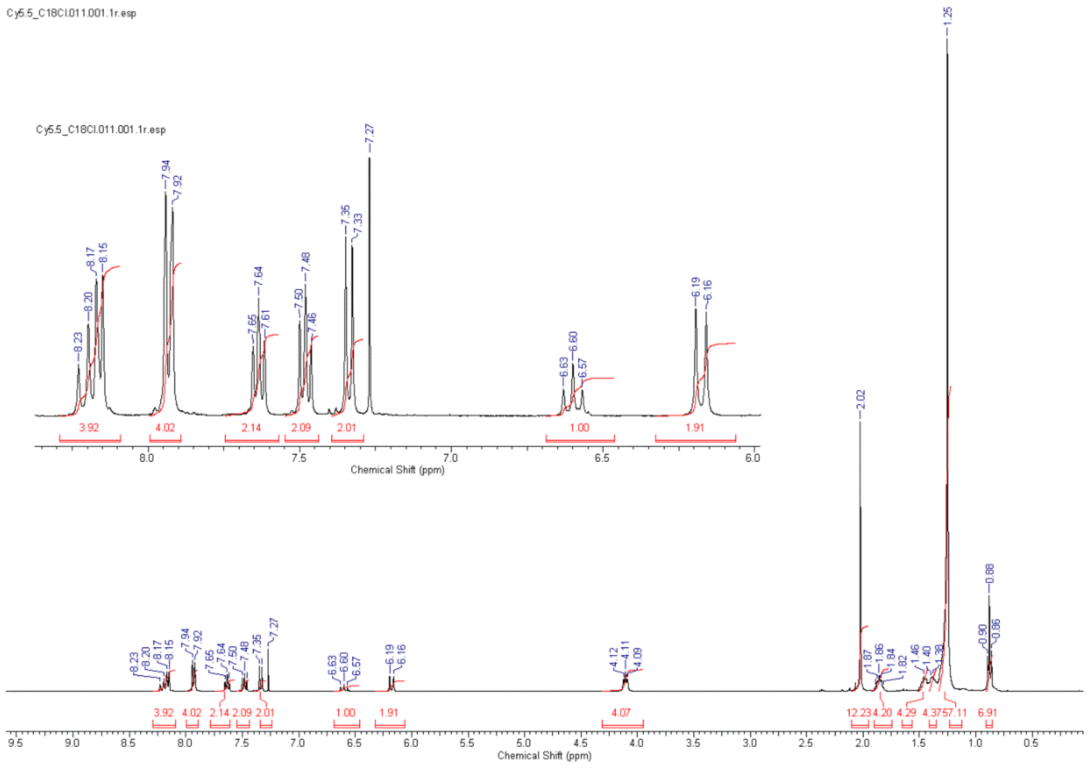

^{1}H-NMR spectrum of Cy5.5 chloride.

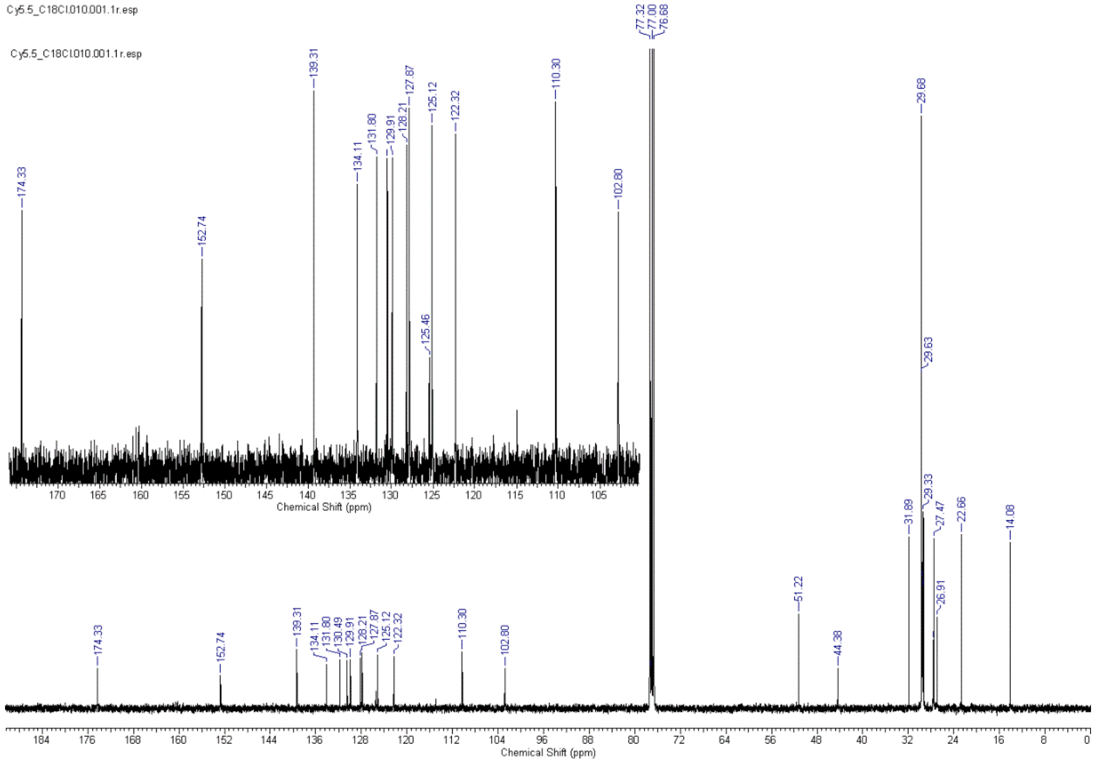

^{13}C-NMR spectrum of Cy5.5 chloride.

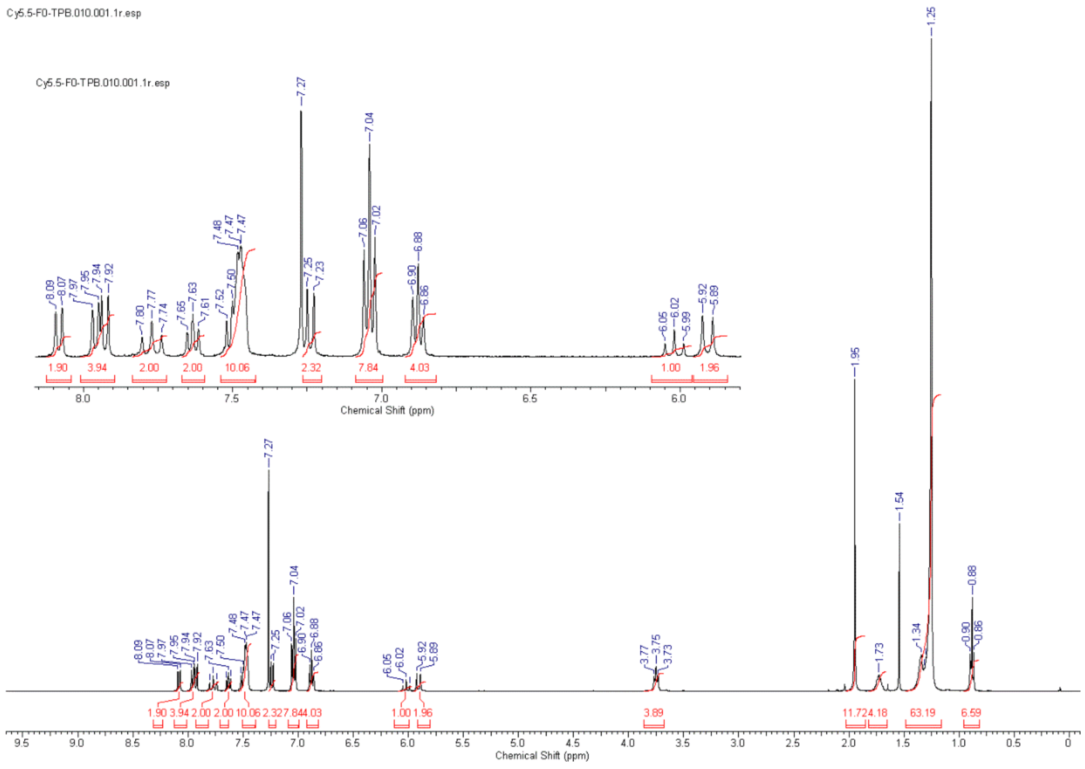

^{1}H-NMR spectrum of Cy5.5/TPB.

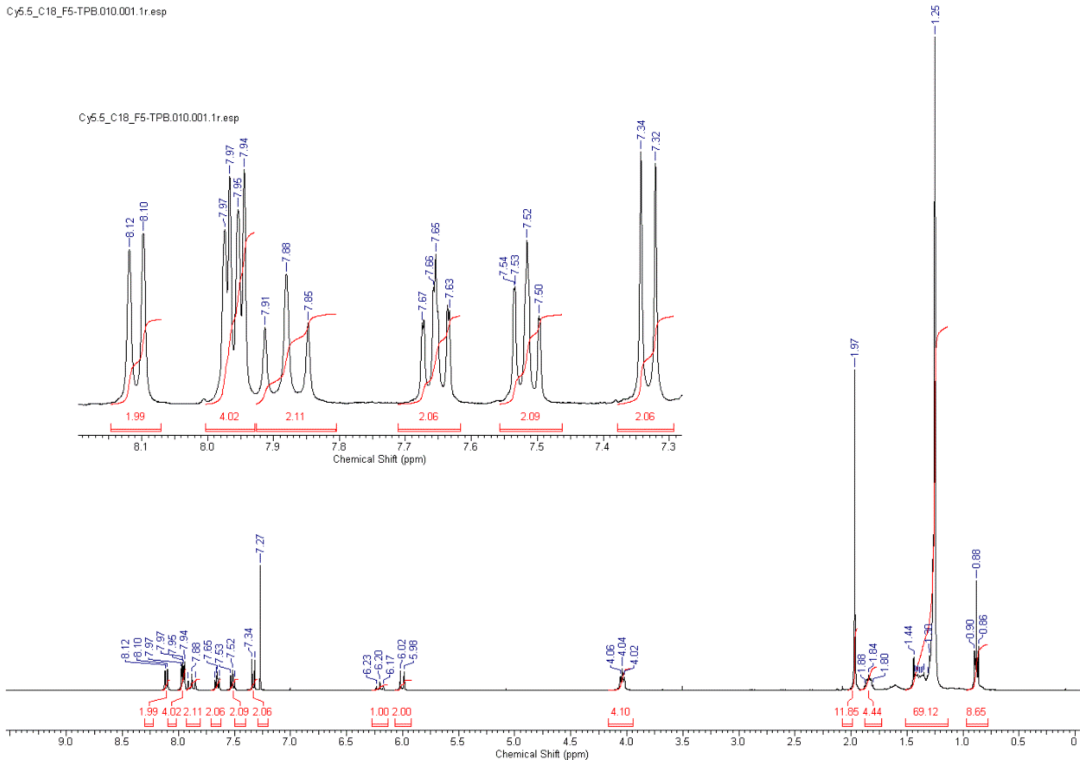

^{1}H-NMR spectrum of Cy5.5/F5-TPB.

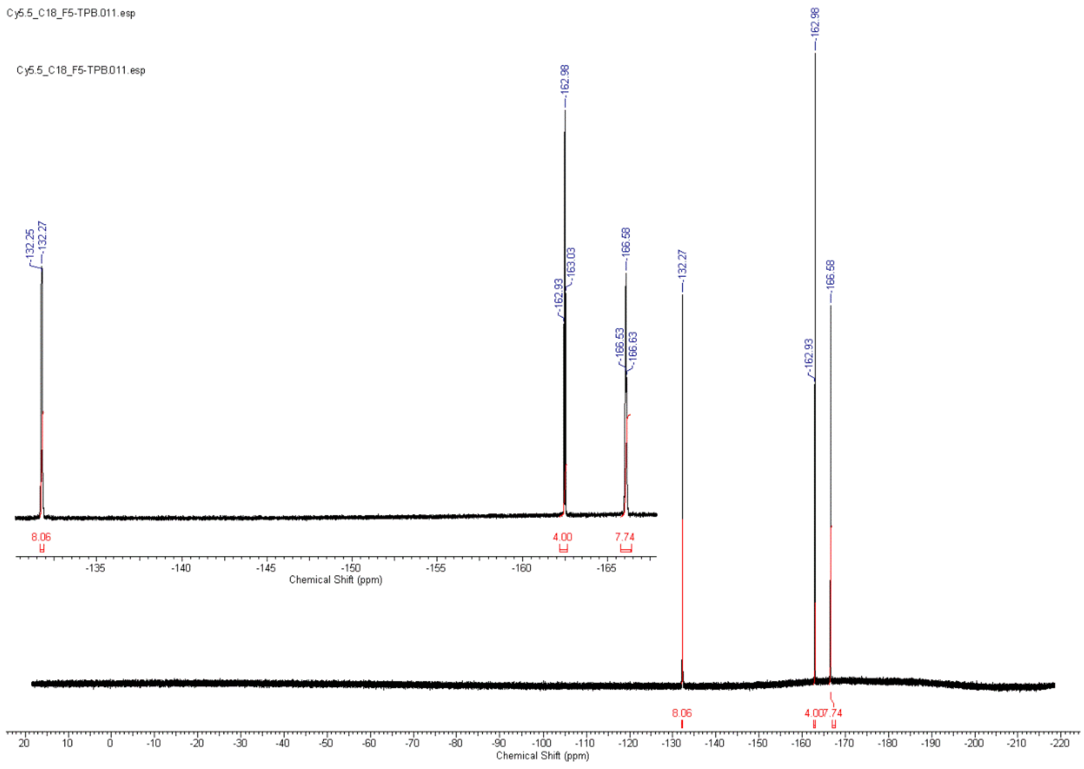

^{19}F-NMR spectrum of Cy5.5/F5-TPB.

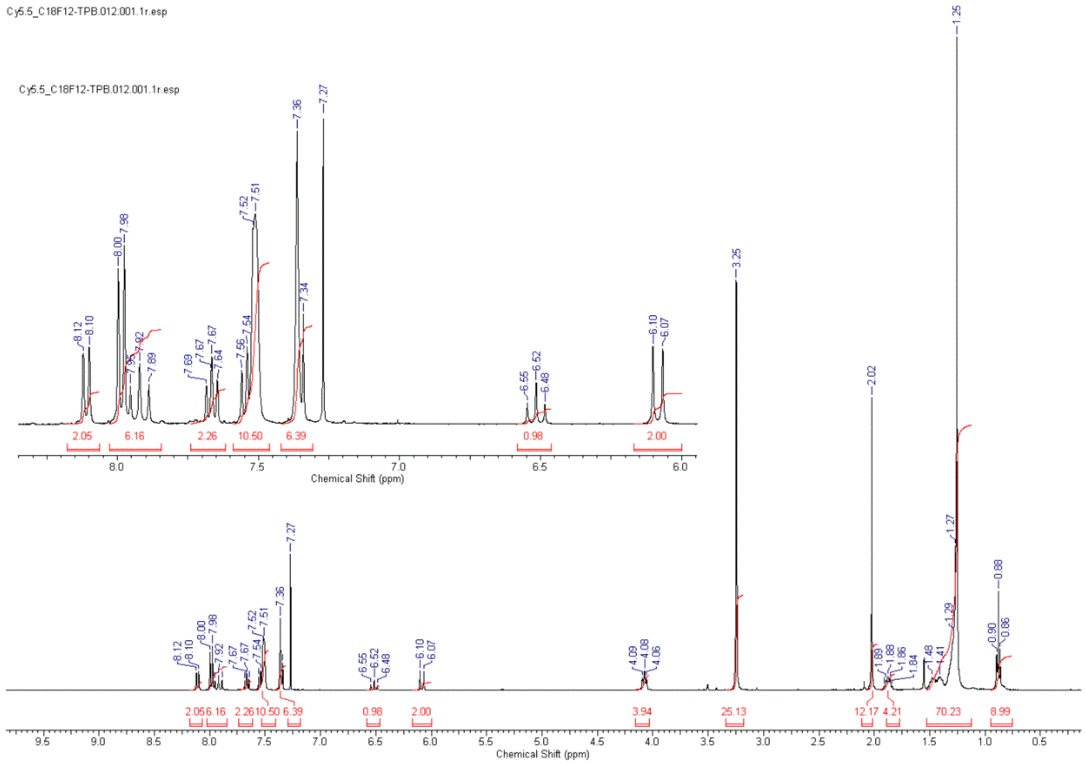

^{1}H-NMR spectrum of Cy5.5/F12-TPB.

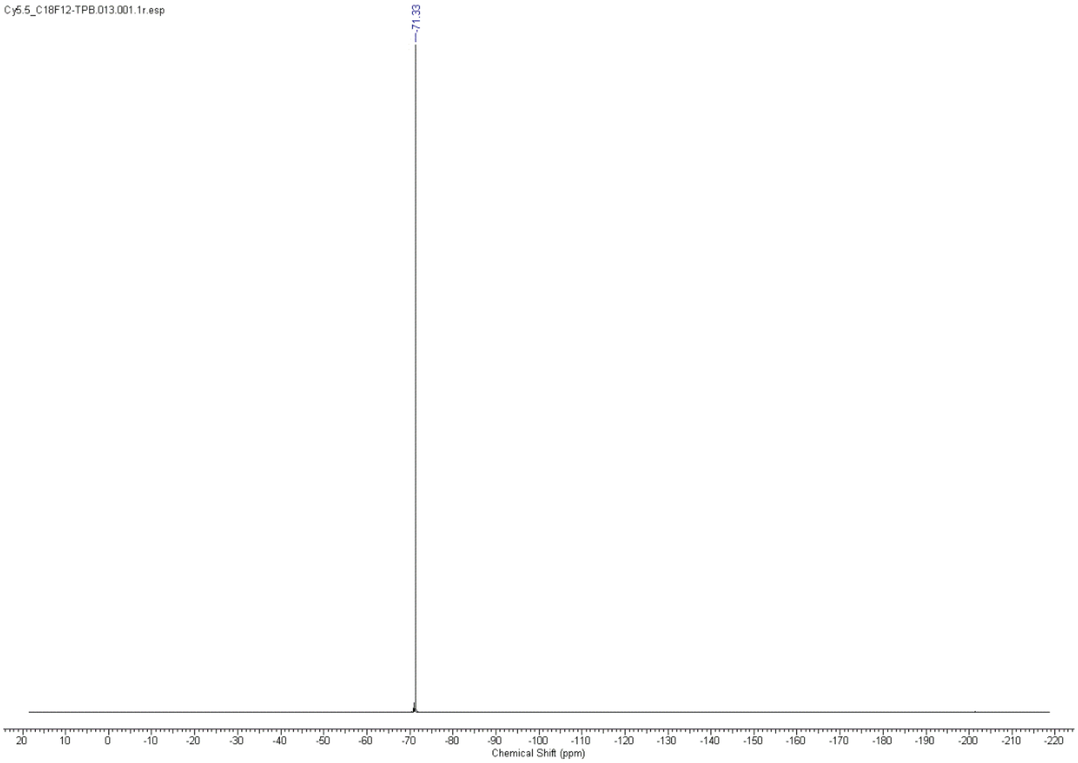

^{19}F-NMR spectrum of Cy5.5/F12-TPB.

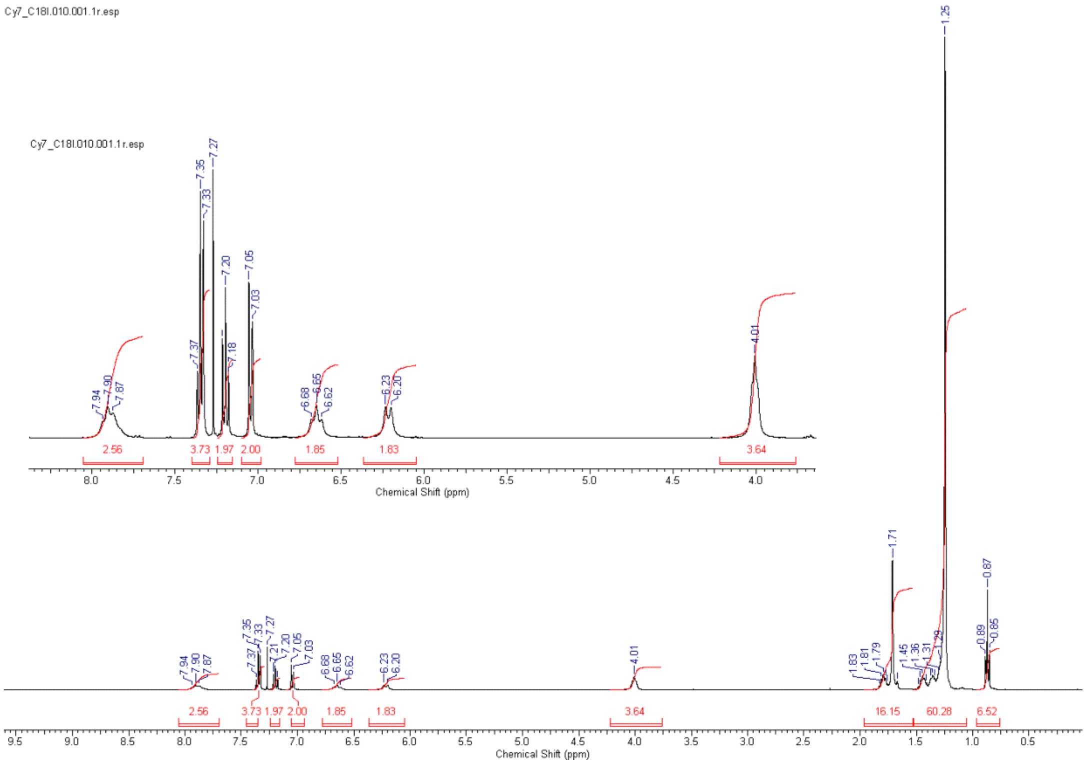

^{1}H-NMR spectrum of Cy7 chloride.

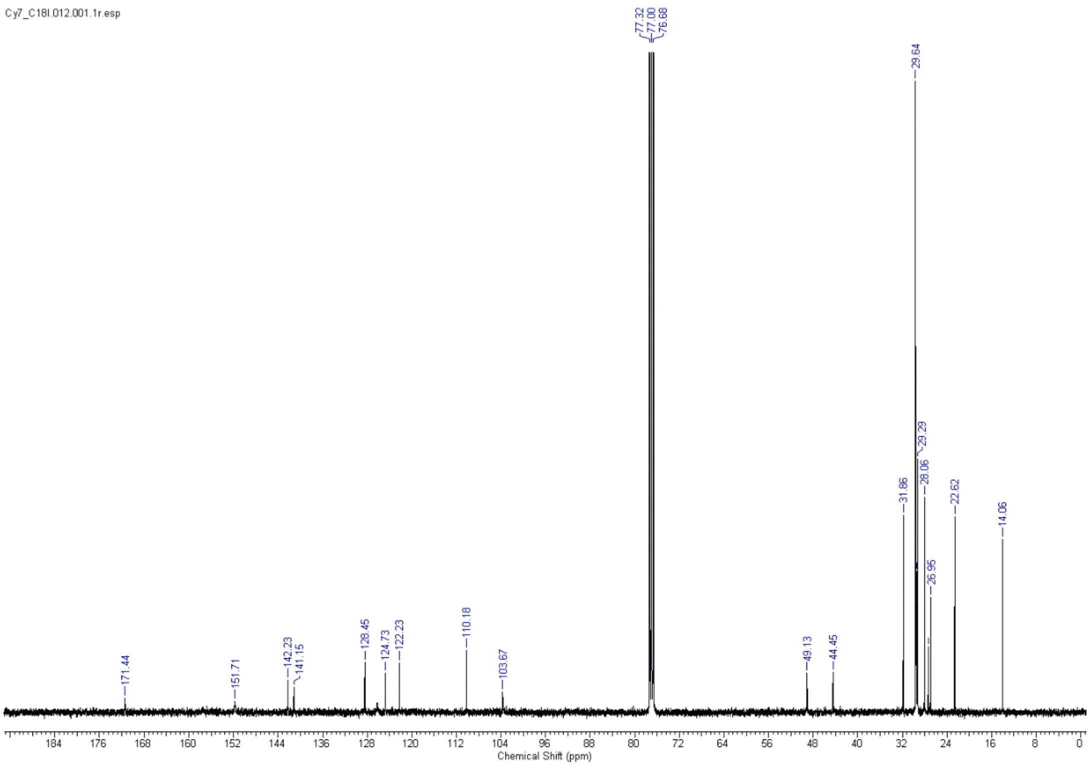

^{13}C-NMR spectrum of Cy7 chloride.

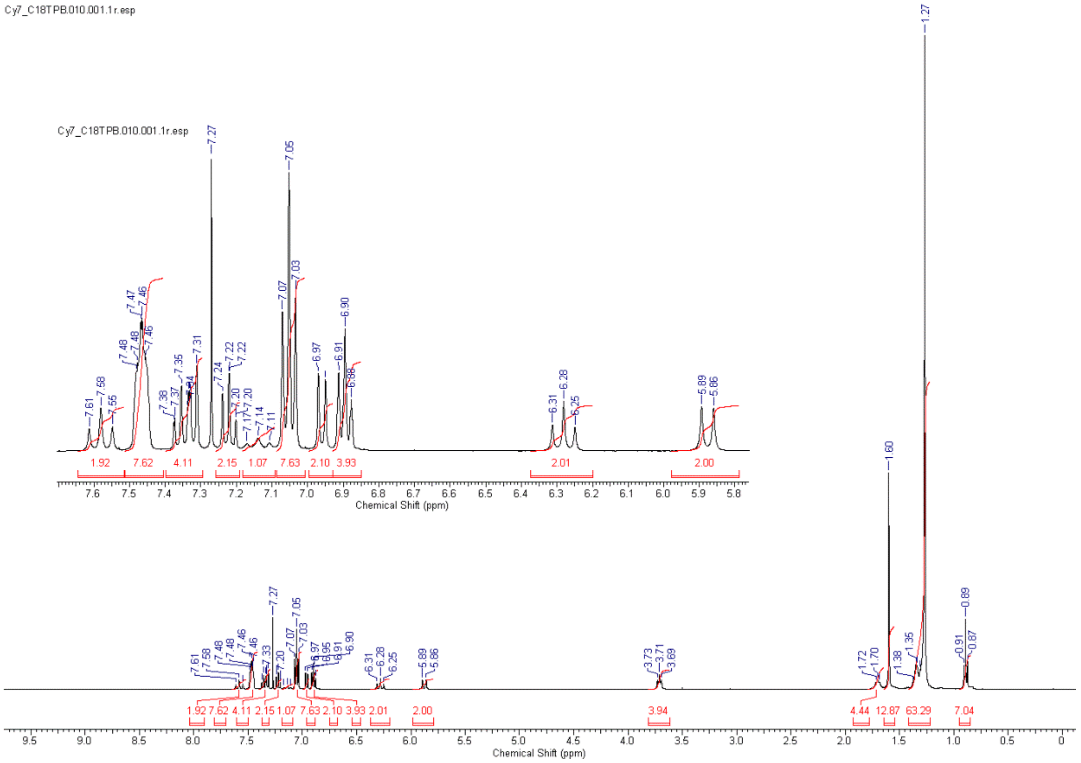

^{1}H-NMR spectrum of Cy7/TPB.

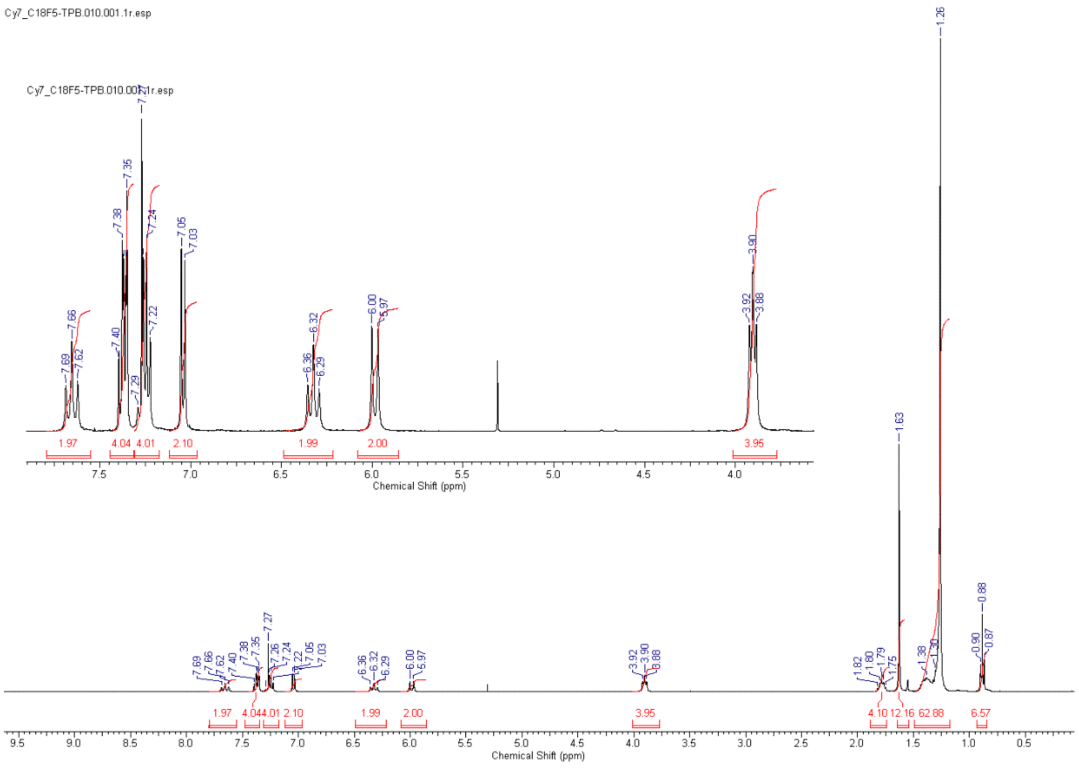

^{1}H-NMR spectrum of Cy7/F5-TPB.

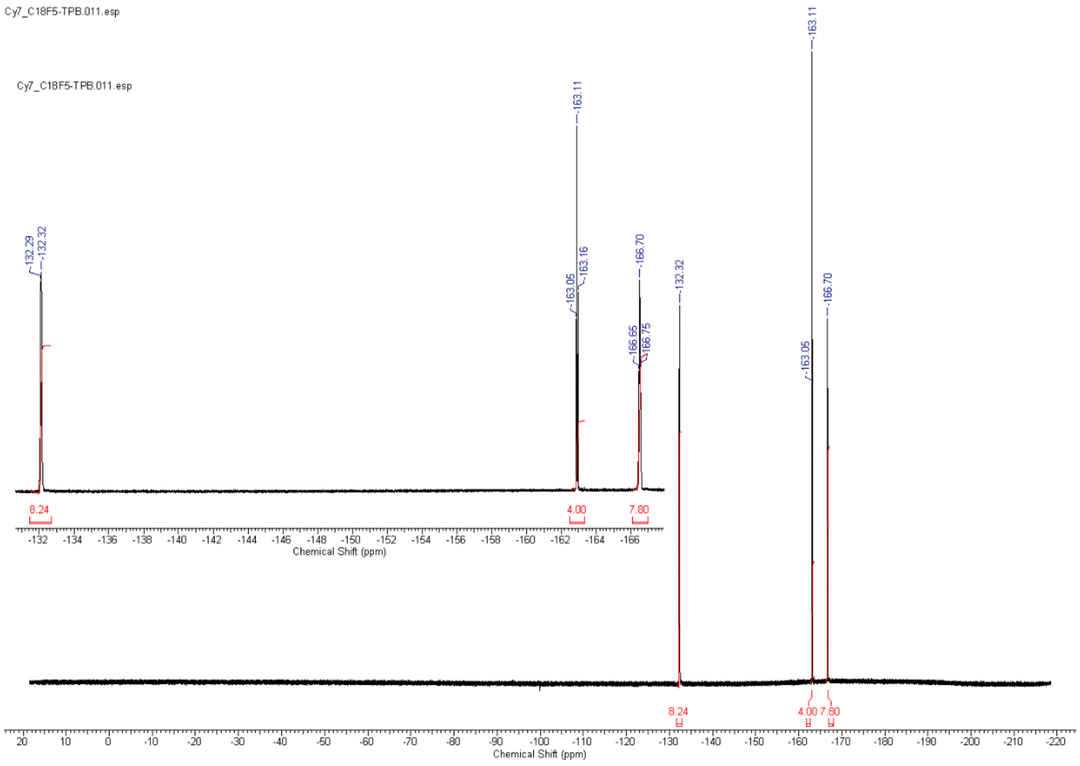

^{19}F-NMR spectrum of Cy7/F5-TPB.

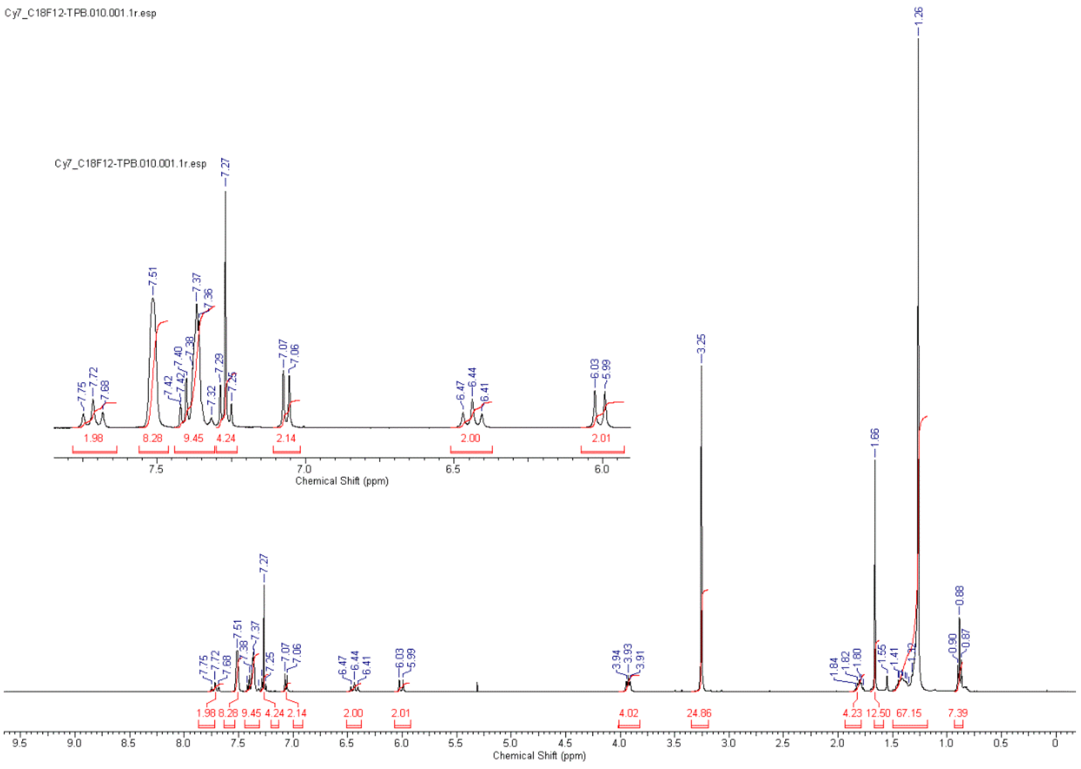

^{1}H-NMR spectrum of Cy7/F12-TPB.

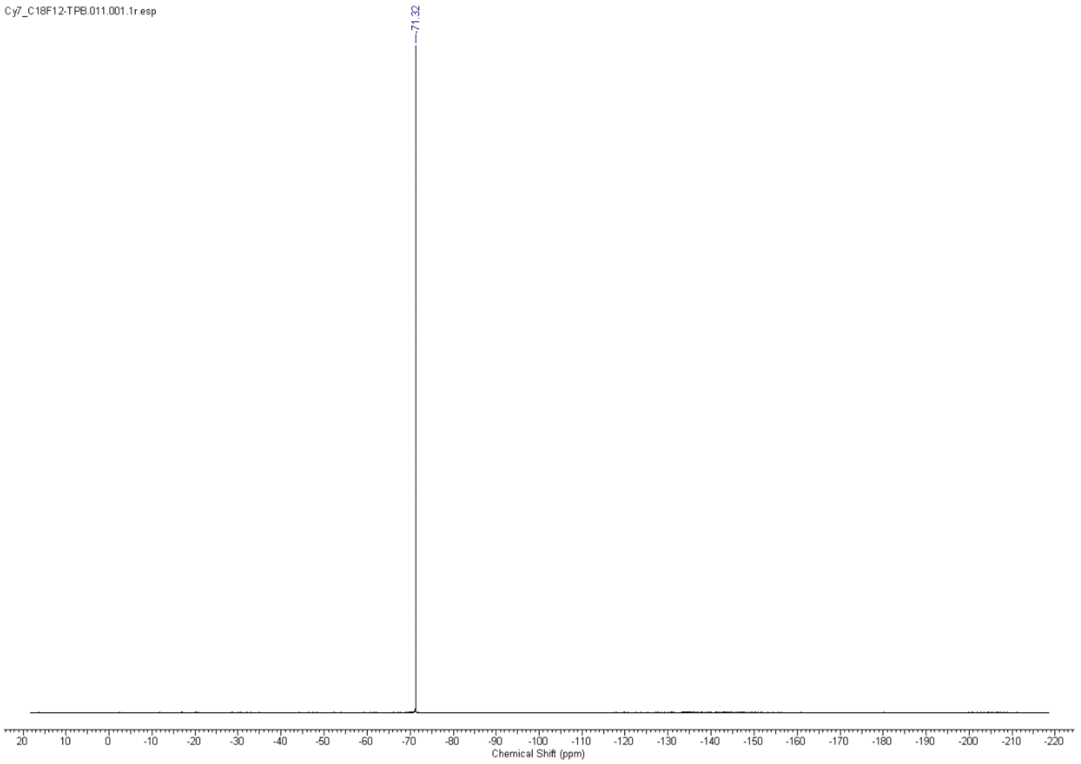

^{19}F-NMR spectrum of Cy7/F12-TPB.
